# Genetic polymorphisms of the *GNRH1 *and *GNRHR *genes and risk of breast cancer in the National Cancer Institute Breast and Prostate Cancer Cohort Consortium (BPC3)

**DOI:** 10.1186/1471-2407-9-257

**Published:** 2009-07-29

**Authors:** Federico Canzian, Rudolf Kaaks, David G Cox, Katherine D Henderson, Brian E Henderson, Christine Berg, Sheila Bingham, Heiner Boeing, Julie Buring, Eugenia E Calle, Stephen Chanock, Francoise Clavel-Chapelon, Laure Dossus, Heather Spencer Feigelson, Christopher A Haiman, Susan E Hankinson, Robert Hoover, David J Hunter, Claudine Isaacs, Per Lenner, Eiliv Lund, Kim Overvad, Domenico Palli, Celeste Leigh Pearce, Jose R Quiros, Elio Riboli, Daniel O Stram, Gilles Thomas, Michael J Thun, Dimitrios Trichopoulos, Carla H van Gils, Regina G Ziegler

**Affiliations:** 1 German Cancer Research Center (DKFZ), Heidelberg, Germany; 2 Harvard School of Public Health, Boston, MA, USA; 3 University of Southern California, Los Angeles, CA, USA; 4 Beckman Research Institute of the City of Hope National Medical Center, Duarte, CA, USA; 5 Division of Cancer Epidemiology and Genetics, National Cancer Institute, Bethesda, MD, USA; 6 MRC Dunn Human Nutrition Unit, Cambridge, UK; 7 Department of Epidemiology, German Institute of Human Nutrition Potsdam-Rehbruecke, Nuthetal, Germany; 8 Harvard Medical School, Boston, MA, USA; 9 American Cancer Society, Atlanta, GA, USA; 10 INSERM, Institut Gustave Roussy, Villejuif, France; 11 Lombardi Comprehensive Cancer Center, Georgetown University, Washington DC, USA; 12 Department of Radiation Sciences, Oncology, Umeå University, Umeå, Sweden; 13 Institute of Community Medicine, University of Tromsø, Tromsø, Norway; 14 Department of Clinical Epidemiology, Aarhus University Hospital, Aalborg, Denmark; 15 Molecular and Nutritional Epidemiology Unit, CSPO-Scientific Institute of Tuscany, Florence, Italy; 16 Public Health and Health Planning Directorate, Asturias, Spain; 17 Imperial College, London, UK; 18 Department of Hygiene and Epidemiology, School of Medicine, University of Athens, Athens, Greece; 19 Julius Center for Health Sciences and Primary Care, University Medical Center, Utrecht, the Netherlands

## Abstract

**Background:**

Gonadotropin releasing hormone (GNRH1) triggers the release of follicle stimulating hormone and luteinizing hormone from the pituitary. Genetic variants in the gene encoding GNRH1 or its receptor may influence breast cancer risk by modulating production of ovarian steroid hormones. We studied the association between breast cancer risk and polymorphisms in genes that code for GNRH1 and its receptor (GNRHR) in the large National Cancer Institute Breast and Prostate Cancer Cohort Consortium (NCI-BPC3).

**Methods:**

We sequenced exons of *GNRH1 *and *GNRHR *in 95 invasive breast cancer cases. Resulting single nucleotide polymorphisms (SNPs) were genotyped and used to identify haplotype-tagging SNPs (htSNPS) in a panel of 349 healthy women. The htSNPs were genotyped in 5,603 invasive breast cancer cases and 7,480 controls from the Cancer Prevention Study-II (CPS-II), European Prospective Investigation on Cancer and Nutrition (EPIC), Multiethnic Cohort (MEC), Nurses' Health Study (NHS), and Women's Health Study (WHS). Circulating levels of sex steroids (androstenedione, estradiol, estrone and testosterone) were also measured in 4713 study subjects.

**Results:**

Breast cancer risk was not associated with any polymorphism or haplotype in the *GNRH1 *and *GNRHR *genes, nor were there any statistically significant interactions with known breast cancer risk factors. Polymorphisms in these two genes were not strongly associated with circulating hormone levels.

**Conclusion:**

Common variants of the *GNRH1 *and *GNRHR *genes are not associated with risk of invasive breast cancer in Caucasians.

## Background

Exposure to steroid hormones (estrogens and androgens) is a risk factor for breast cancer. Increased exposure to estrogens, for instance by early menarche, late menopause, low parity and post-menopausal obesity, contributes to increased breast cancer risk (reviewed in ref. [1]). High circulating levels of estrogens are associated with elevated breast cancer risk [2,3].

The primary stimulus for production of estrogen and other ovarian steroid hormones is the release of the gonadotropins, follicle-stimulating hormone (FSH) and luteinizing hormone (LH), from the anterior pituitary. These are released when gonadotropin-releasing hormone 1 (GNRH1), from the hypothalamus, binds to the gonadotropin-releasing hormone receptor (GNRHR) in the anterior pituitary. The resultant G-protein activation of a phosphatidylinositol-calcium second messenger system ultimately triggers the release of FSH and LH.

GNRH1 activity is low during childhood but increases at puberty. GNRH1 production is pulsatile. In females, the GNRH1 pulse frequency varies during the menstrual cycle, with a large surge of GNRH1 just before ovulation. The size and frequency of the GNRH1 pulse, and feedback from androgens and estrogens, control production of LH and FSH [4].

GNRH1 activity can be disrupted by hypothalamic-pituitary disease. Elevated prolactin levels decrease GNRH1 activity. In contrast, hyperinsulinemia increases pulse activity leading to disorderly LH and FSH activity, as seen in polycystic ovary syndrome [5].

The *GNRH1 *gene is located on chromosome 8p21.2. It spans about 5 kb and contains 3 exons. It encodes the GNRH1 precursor, which contains 92 amino acids and is processed to GNRH1, a decapeptide. The *GNRHR *gene is located on chromosome 4q13.2. Its genomic sequence covers about 19 kb and it includes 3 exons.

The *GNRH1 *and *GNRHR *genes can harbor rare germline mutations which lead to idiopathic hypogonadotropic hypogonadism (IHH) or Kallmann syndrome (MIM 146110, 147950) in both men and women [6]. Common variants have not been studied for either gene in relation to cancer risk.

We hypothesized that common, functional polymorphisms of *GNRH1 *and *GNRHR *could influence breast cancer risk by modifying production of FSH/LH and steroid hormones. We used a haplotype tagging approach to examine this hypothesis using cases and controls from the BPC3.

## Methods

### Study Population

The BPC3 has been described in detail elsewhere [7]. Briefly, the consortium includes large, well-established cohorts assembled in the United States and Europe, that have both DNA samples and extensive questionnaire information. These include: the American Cancer Society Cancer Prevention Study II (CPS-II) [8], the European Prospective Investigation into Cancer and Nutrition (EPIC) [9], the Harvard Nurse's Health Study (NHS) [10] and Women's Health Study (WHS) [11], and the Multiethnic Cohort (MEC) [12].

Cases were identified in each cohort by self report with subsequent confirmation of the diagnosis from medical records or tumor registries, and/or linkage with population-based tumor registries (method of confirmation varied by cohort). Controls were matched to cases by ethnicity and age, and in some cohorts, additional criteria, such as country of residence in EPIC.

Most of the subjects were Caucasians of European descent. One cohort (MEC) provided most of the non-Caucasian samples. In total, we genotyped 4,401 Caucasian cases and 5,966 controls, 329 Latino cases and 385 controls, 341 African American cases and 426 controls, 425 Japanese American cases and 418 controls, and 107 Native Hawaiian cases and 285 controls.

Written informed consent was obtained from all subjects, and the project has been approved by the competent institutional review boards for each cohort.

### Selection of haplotype tagging single nucleotide polymorphisms (htSNPs)

We sequenced exons and intron/exon junctions of *GNRH1 *and *GNRHR *in a panel of 95 metastatic breast cancer cases from the MEC and EPIC. These included 19 cases from each ethnic group represented in the study (African American, Latino, Japanese, Native Hawaiian, and Caucasian). About 45 kb were surveyed for *GNRH1 *and about 56 kb for *GNRHR*. No non-synonymous or splice-site variants were identified in sequencing of the exons.

Based on the resequencing and SNPs available in dbSNP, we identified 17 SNPs in GNRH1 and 36 SNPs in GNRHR with minor allele frequency greater than 5% in any of the five ethnic groups or greater than 1% overall. These SNPs were genotyped in a reference panel of 349 healthy women (70 African-Americans, 68 Latinos, 72 Japanese, 70 Caucasians, and 69 Hawaiians from the MEC cohort who had not been diagnosed with breast cancer at the time of the study; average age 65.1 (standard deviation 8.5)) at the Broad Institute (Cambridge, MA, USA) using the Sequenom (San Diego, CA, USA) and Illumina (San Diego, CA, USA) platforms.

Haplotype tagging SNPs (htSNPs) were then selected using the method of Stram *et al*. [13] to maximize R^2^_H _among Caucasians. Three htSNPs were selected for *GNRH1 *(including one localized in the 5' neighboring gene, *KCTD9*, and one in the gene at the 3', *DOCK5*) and seven for *GNRHR*.

### Genotyping

Genotyping of htSNPs was performed in 3 laboratories (University of Southern California, Los Angeles, CA, USA; Harvard School of Public Health, Boston, MA, USA; International Agency for Research on Cancer, Lyon, France) using a fluorescent 5' endonuclease assay and the ABI-PRISM 7900 for sequence detection (TaqMan). Initial quality control checks of the SNP assays were performed by the manufacturer (Applied Biosystems, Foster City, CA, USA); an additional 500 test reactions were run at the University of Southern California. Characteristics for the 10 TaqMan assays are available on a public website http://www.uscnorris.com/mecgenetics/CohortGCKView.aspx. Sequence validation for each SNP assay was performed on samples from the SNP500 project http://snp500cancer.nci.nih.gov[14] and 100% concordance was observed. To assess inter-laboratory variation, each genotyping center ran assays on a designated set of 94 samples from the Coriell Biorepository (Camden, NJ, USA) included in SNP500. The internal quality of genotype data at each genotyping center was assessed by typing 5–10% blinded samples in duplicate or triplicate (depending on study).

### Hormone Analysis

Circulating serum hormones were measured at the International Agency for Research on Cancer for EPIC and MEC samples and at the Harvard School of Public Health for NHS samples, for a total of 4713 subjects (1405 cases and 3308 controls, 1120 pre-menopausal and 3593 post-menopausal subjects). The different assays for hormone analyses were chosen on the basis of a previously published comparative validation study [15]. Estradiol (E_2_), estrone (E_1_) and androstenedione (Δ_4_) were measured by direct double-antibody radioimmunoassays from DSL (Diagnostic Systems Laboratories, Texas), while testosterone (T) was measured by direct radioimmunoassays from Immunotech (Marseille, France). Measurements were performed on never thawed serum sample aliquots. Mean intrabatch and interbatch coefficients of variation were 5.8 and 13.1%, respectively, for E_2 _(at a concentration of 250 pmol/l), 10.2 and 12.6% for E_1 _(at 75 pmol/l), and 4.8 and 18.9% for Δ_4 _(at 1.40 nmol/l), 10.8 and 15.3% for T (at 1.40 nmol/l).

### Statistical Analysis

We used conditional multivariate logistic regression to estimate odds ratios (ORs) for invasive breast cancer in subjects with a linear (log-odds additive) scoring for 0, 1 or 2 copies of the minor allele of each SNP. We also used conditional logistic regression with additive scoring and the most common haplotype as the referent to estimate haplotype-specific ORs using an expectation-substitution approach to assign haplotypes based on the unphased genotype data and to account for uncertainty in assignment [16,17]. Haplotype frequencies and expected subject-specific haplotype indicators were calculated separately for each cohort (and country within EPIC or ethnicity in the MEC). We combined rare haplotypes (those with estimated individual frequencies less than 3% in all cohorts) into a single category, which had a combined frequency of less than 1% of the controls for both genes and both linkage disequilibrium (LD) blocks of *GNRHR*. To test the global null hypothesis of no association between variation in *GNRH1*/*GNRHR *haplotypes and htSNPs and risk of invasive breast cancer (or subtypes defined by receptor status), we used a likelihood ratio test comparing a model with additive effects for each common haplotype (treating the most common haplotype as the referent) to the intercept-only model.

We performed subgroup analyses stratifying by cohort, ethnicity, country within EPIC, estrogen receptor/progesterone receptor status, metastatic *vs*. localized disease, and age at diagnosis (≤55 years *vs*. >55 years). We also investigated interactions between single SNPs or haplotypes and completion of a full term pregnancy (ever/never), age at first full term pregnancy (in three categories: nulliparous, ≤24, >24), body mass index (BMI in kg/m^2 ^in three categories: <25, 25–29, ≥30), height (<160 cm, 160–165 cm, >165 cm), smoking status (never/former/current smoker), and use of menopausal hormone therapy (ever/never). Other common risk factors, including family history of breast cancer, personal history of benign breast disease, and age at menopause were unavailable for large numbers of women, and therefore were not included in the models.

Relationships of genetic variants with serum hormone levels were estimated by standard regression models, adjusted for BMI, age, assay batch, ethnicity, and country within EPIC. These analyses were performed both using all the study subjects for whom hormone levels have been measured, and only the controls, who represent the populations giving rise to the cases.

## Results

**Figure 1:**
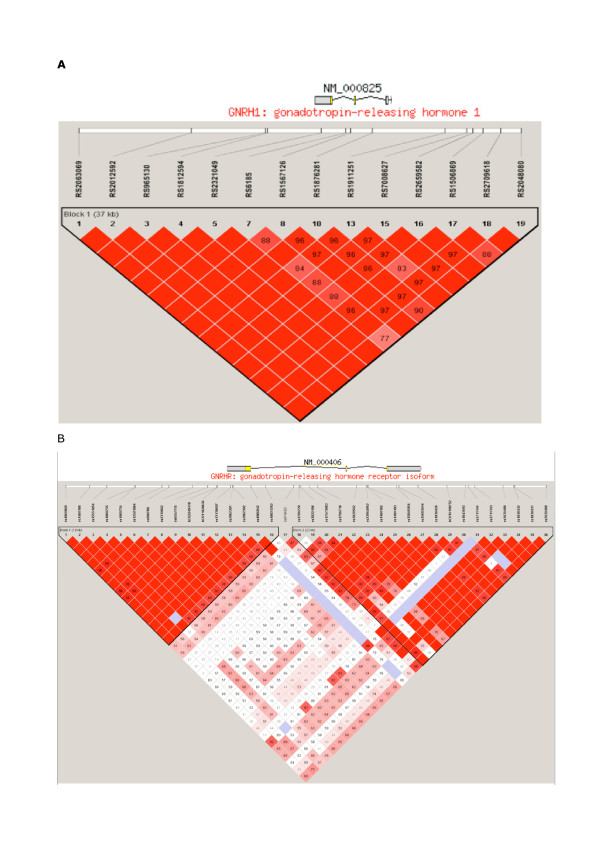
**Haploview plot of the genomic region of *GNRH1 *(A) and *GNRHR *(B)**. From top to bottom: position of genes (boxes: exons, lines: introns), SNPs genotyped in the multiethnic panel, graphical representation of LD and block structure (darker color represents higher LD, numbers in the colored squares are percentage of LD, expressed as D', absence of number means D' = 100%).

The genomic regions surrounding *GNRH1 *and *GNRHR *are shown in Figure 1. *GNRH1 *consists of a single LD block, whereas *GNRHR *includes two LD blocks, one of them including exon 1 and the other exons 2 and 3. *GNRH1 *was tagged by 3 SNPs, which account for 94% of haplotype diversity. Block 1 of *GNRHR *was tagged by 3 SNPs and block 2 by 4 SNPs (98% and 95% of haplotype diversity, respectively). Frequency of common haplotypes ranged between 19 and 35% in controls for *GNRH1 *and 5% and 52% for *GNRHR*.

A total of 5,603 invasive breast cancer cases and 7,480 controls were available for genotyping from each of the participating cohorts. Samples not yielding a genotype were removed from individual SNP analyses, and samples not yielding at least one genotype were removed from haplotype analyses. Both between-center genotyping concordance and within-center blinded quality control concordance were above 99%. Genotype success rate among cases and controls in all cohorts was greater than 95%. No polymorphisms deviated from Hardy-Weinberg Equilibrium among the controls.

Detailed results of associations between serum concentrations of steroid hormones and SNPs are presented in Additional file 1. SNP rs2630488 within *GNRHR *showed a nominally significant association (p = 0.04) with estradiol levels in post-menopausal women. Presence of the minor allele at this polymorphism was associated with an increase in estradiol level (a 4% increase among homozygotes for the minor allele, as compared to homozygotes for the common allele). However, this effect was entirely driven by the association observed in post-menopausal breast cancer cases within EPIC (p = 0.012 in this subgroup), and was not observed in the other subgroups. A few borderline associations were observed between hormone levels and SNPs in pre-menopausal women, however sample size of this group of subjects was considerably smaller, and all subjects derived from only one cohort (EPIC). No associations between polymorphisms and hormone levels remained significant after correction for multiple testing.

Results of association analyses between htSNPs of *GNRH1 *and breast cancer risk are presented in Table 1, and results of haplotype analysis in Table 2. Results of analyses for *GNRHR *are presented in Tables 3 and 4. No association was observed for any of the htSNPs of either gene. Haplotype analysis also showed no association, with global tests for comparison of haplotypes frequency in cases and controls resulting in non-significant results (Wald tests: d.f. = 4, p = 0.364 for *GNRH1*; d.f. = 5, p = 0.897 for *GNRHR *block 1 and d.f. = 6, p = 0.967 for *GNRHR *block 2). Analyses were unadjusted (conditional on matching criteria) or adjusted for known breast cancer risk factors, but results did not show any difference.

**Table 1:** Association between *GNRH1 *htSNPs and breast cancer risk in the BPC3 study.

SNP	Genotype	Cases (%)	Controls (%)	OR (95% CI)^a^	p value
rs2709618	G/G	2,094 (38)	2,771 (38)	1.00 (ref.)	
	G/A	2,547 (46)	3,412 (46)	1.00 (0.93–1.08)	0.942
	A/A	868 (16)	1,163 (16)	1.00 (0.90–1.10)	0.927
					p_trend _= 0.958
rs6185	C/C	3,023 (56)	4,036 (56)	1.00 (ref.)	
	C/G	1,963 (36)	2,684 (37)	0.98 (0.91–1.05)	0.597
	G/G	418 (8)	545 (8)	0.99 (0.87–1.13)	0.889
					p_trend _= 0.695
rs1812594	T/T	3,424 (64)	4,565 (65)	1.00 (ref.)	
	T/C	1,730 (32)	2,158 (31)	1.08 (1.00–1.16)	0.040
	C/C	203 (4)	285 (4)	0.98 (0.82–1.17)	0.825
					p_trend _= 0.156

^a^Odds ratios and 95% confidence intervals, calculated by unadjusted logistic regression analysis conditional on the matching variables

**Table 2:** Association between *GNRH1 *haplotypes and breast cancer risk in the BPC3 study.

Haplotype	Cases (%)	Controls (%)	OR (95% CI)^a^	p value
hGCT	2,024 (35)	2,697 (35)	1.00 (ref.)	
hGGT	1,469 (26)	1,955 (26)	1.00 (0.94–1.06)	0.9480
hACC	1,137 (20)	1,487 (19)	1.04 (0.97–1.11)	0.2957
hACT	1,074 (19)	1,484 (19)	0.96 (0.90–1.03)	0.2240
Freq<3%	25 (<1)	34 (<1)	0.87 (0.58–1.31)	0.5056

^a^Odds ratios and 95% confidence intervals, calculated by unadjusted logistic regression analysis conditional on the matching variables

**Table 3:** Association between *GNRHR *htSNPs and breast cancer risk in the BPC3 study.

SNP	Genotype	Cases (%)	Controls (%)	OR (95% CI)^a^	p value
rs13138607	G/G	1,413 (26)	1,948 (27)	1.00 (ref.)	
	G/A	2,696 (49)	3,550 (48)	1.03 (0.95–1.11)	0.484
	A/A	1,380 (25)	1,829 (25)	1.01 (0.92–1.11)	0.789
					p_trend _= 0.781
rs4986942	G/G	4,650 (84)	6,200 (84)	1.00 (ref.)	
	G/A	867 (16)	1,136 (15)	1.04 (0.95–1.14)	0.350
	A/A	34 (1)	41 (1)	0.99 (0.66–1.49)	0.967
					p_trend _= 0.402
rs10031252	T/T	1,503 (27)	2,093 (28)	1.00 (ref.)	
	T/A	2,694 (49)	3,566 (48)	1.02 (0.94–1.10)	0.640
	A/A	1,324 (24)	1,701 (23)	1.02 (0.93–1.12)	0.641
					p_trend _= 0.637
rs3822196	A/A	3,259 (59)	4,202 (58)	1.00 (ref.)	
	A/G	1,872 (34)	2,589 (36)	0.96 (0.89–1.03)	0.223
	G/G	350 (6)	480 (7)	0.97 (0.8–41.11)	0.622
					p_trend _= 0.273
rs3796718	T/T	2,951 (55)	3,868 (54)	1.00 (ref.)	
	T/C	2,053 (38)	2,761 (38)	0.98 (0.92–1.05)	0.642
	C/C	409 (8)	571 (8)	0.96 (0.84–1.09)	0.497
					p_trend _= 0.461
rs1843593	T/T	4,012 (73)	5,277 (72)	1.00 (ref.)	
	T/C	1,357 (25)	1,843 (25)	1.01 (0.93–1.09)	0.831
	C/C	122 (2)	188 (3)	0.98 (0.79–1.22)	0.848
					p_trend _= 0.942
rs2630488	A/A	1,530 (29)	1,959 (27)	1.00 (ref.)	
	A/G	2,618 (49)	3,489 (49)	1.00 (0.92–1.08)	0.942
	G/G	1,200 (22)	1,694 (24)	0.96 (0.88–1.06)	0.436
					p_trend _= 0.458

^a^Odds ratios and 95% confidence intervals, calculated by unadjusted logistic regression analysis conditional on the matching variables

**Table 4:** Association between *GNRHR *haplotypes and breast cancer risk in the BPC3 study.

Haplotype	Cases (%)	Controls (%)	OR (95% CI)^a^	p value
Block 1				
hAGA	2,426 (43)	3,161 (42)	1.00 (ref.)	
hGGT	2,060 (36)	2,806 (37)	0.98 (0.93–1.03)	0.491
hGAT	473 (8)	620 (8)	1.02 (0.93–1.11)	0.712
hAGT	397 (7)	557 (7)	0.96 (0.87–1.06)	0.433
hGGA	307 (5)	400 (5)	0.97 (0.87–1.07)	0.522
Freq<3%	4 (<1)	6 (<1)	0.83 (0.31–2.25)	0.720
				
Block 2				
hATTA	2,997 (53)	3,908 (52)	1.00 (ref.)	
hGCTG	1,178 (21)	1,630 (22)	0.99 (0.92–1.06)	0.342
hATCG	667 (12)	937 (12)	1.00 (0.92–1.09)	0.731
hATTG	478 (8)	633 (8)	1.01 (0.89–1.14)	0.949
hACTG	177 (3)	214 (3)	0.98 (0.85–1.15)	0.862
hGCCG	150 (3)	206 (3)	1.11 (0.76–1.62)	0.836
Freq<3%	21 (<1)	22 (<1)	0.98 (0.93–1.03)	0.579

^a^Odds ratios and 95% confidence intervals, calculated by unadjusted logistic regression analysis conditional on the matching variables

Analyses performed by stratifying cases by age at diagnosis (greater or lower than 55 years), localized or metastatic disease or estrogen/progesterone receptor status did not show significant differences. Stratification of subjects by cohort, country in EPIC or ethnicity in MEC showed only few results supported by p values ranging from 0.01 to 0.05, which were always based on a small number of subjects. For these, we performed heterogeneity tests, which in all cases were not statistically significant. For example, heterozygotes for SNP rs1812594 of GNRH1 had an odds ratio (OR) of 1.08 (95% confidence interval (CI) = 1.00–-1.16, p = 0.04). When we analyzed the results for each cohort, it resulted that the association was driven by EPIC data (OR = 1.16, 95% CI 1.00–1.34, p = 0.046), and within EPIC the only significant result came from the Spanish sub-cohort (OR = 1.59, 95% CI = 1.07–2.37, p = 0.021), which is based on 62 cases and 79 controls heterozygous for this SNP. However, heterogeneity tests for this genotype were not statistically significant for either the entire study (p = 0.226) or within EPIC (p = 0.147). Nor were homozygotes for the less common allele at this SNP significantly associated with increased risk in any subgroup.

No statistically significant interactions were observed between htSNPs or haplotypes and known breast cancer risk factors, including age at first full term pregnancy, number of pregnancies, never/ever menopausal hormone therapy, height, smoking status, or body mass index.

## Discussion

This large, comprehensive study found no statistically significant associations between polymorphisms in the genes that code for GNRH1 or its receptor and either circulating ovarian sex hormones or breast cancer risk. An influence of SNPs in these two genes on breast cancer risk, mediated by altered levels of estrogens, was plausible, due to the known physiology of steroid hormone stimulation. If any common variants with functional relevance exist in the two candidate genes, our resequencing and haplotype tagging approaches should have detected its effect on hormone measurements and/or cancer risk. The null results are especially convincing because of the large sample size (more than 5,600 invasive breast cancer cases and 7,400 controls) and the extensive resequencing that preceded and informed the selection of htSNPs. The study has over 80% power to detect main effects of common polymorphisms (minor allele frequency of 5% or greater) with relative risks of 1.2 or greater, and to investigate interactions between genetic variants and known environmental or lifestyle exposures [18].

Based on these results, we conclude that common polymorphisms in *GNRH1 *and *GNRHR *do not substantially affect breast cancer risk in Caucasians. Among the many tests performed in subgroups, some associations were supported by p values ranging from 0.01 and 0.05, yet these associations were driven by subgroups containing small numbers of cases, and are therefore compatible with chance. None of these subgroup findings remain statistically significant when adjusted for multiple hypothesis testing. Likewise, the weakly significant association we observed between estradiol levels in post-menopausal women and SNP rs2630488 of *GNRHR *derived from a single subgroup in one cohort, and is also likely to reflect chance.

A limitation of this study was the relatively small number of subjects from racial or ethnic groups other than Caucasian. The MEC provided most of the cases and controls in this regard, but none of these groups exceeded 425 breast cancer cases. This limitation is particularly relevant to African Americans, for whom additional SNPs would be needed to provide comparable coverage of common variants. Coverage is satisfactory for the other ethnic groups [19]. This is consistent with genome-wide data [20], which also showed that tagging SNPs for Caucasians offer good coverage in other ethnic groups, except Africans.

## Conclusion

In conclusion, we can exclude the possibility that common polymorphisms in *GNRH1 *and *GNRHR *confer large or even moderate breast cancer risks in Caucasians. We cannot exclude the possible existence of moderate risks due to polymorphisms of *GNRH1 *and *GNRHR *in non-Caucasian populations. Larger studies of non-Caucasians will be necessary to test this hypothesis.

## Competing interests

The authors declare that they have no competing interests.

## Authors' contributions

FC, RK, DGC, KDH and BEH made up the writing committee for this work, and were responsible for data analyses, manuscript preparation and editing. CLP performed the htSNP selection and contributed substantially to manuscript editing. LD, CAH, DOS, SC provided expertise in genotyping and results analyses, as well as manuscript editing. All other authors contributed substantially to sample collection and manuscript editing. All authors read and approved the final version of the manuscript.

## Pre-publication history

The pre-publication history for this paper can be accessed here:

http://www.biomedcentral.com/1471-2407/9/257/prepub

## Supplementary Material

Additional file 1 Detailed results of associations between serum concentrations of steroid hormones and SNPs. Click here for file
